# Supplementary material for: CCAFE: Estimating Case and Control Allele Frequencies from GWAS Summary Statistics
Source: bioRxiv. 2024 Oct 29:2024.10.24.619530. Preprint. [Version 1] doi: 10.1101/2024.10.24.619530 (PMC11565872; doi:10.1101/2024.10.24.619530)
Supplement: Supplement 1 [file media-1.pdf]

Supplement

Full derivation:

Known:

$$AF_{total} = \frac{(N_{case}AF_{case} + N_{control}AF_{control})}{N_{total}} \quad (1)$$

$$OR = \frac{ad}{bc} \quad (2)$$

Where:

$a = 2N_{case} * AF_{case}$
$b = 2N_{case}(1 - AF_{case})$
$c = 2N_{control} * AF_{control}$
$d = 2N_{control}(1 - AF_{control})$

Find $AF_{control}$ and AF_{case}:

Substitute for $a, b, c, \& \ d$ into equation (2) and simplify:

$$OR = \frac{(2N_{case} * AF_{case})[2N_{control}(1 - AF_{control})]}{[2N_{case}(1 - AF_{case})](2N_{control} * AF_{control})} \quad (3)$$

$$OR = \frac{AF_{case}(1 - AF_{control})}{(1 - AF_{case})AF_{control}} \quad (4)$$

Substitute for AF_{case} using equation (1) and distribute:

$$OR = \frac{(\frac{N_{total}}{N_{case}}AF_{total} - \frac{N_{control}}{N_{case}}AF_{control})(1 - AF_{control})}{[1 - \frac{N_{total}}{N_{case}}AF_{total} + \frac{N_{control}}{N_{case}}AF_{control}]AF_{control}} \quad (5)$$

$$OR = \frac{\frac{N_{total}}{N_{case}}AF_{total} - AF_{control}\frac{N_{control}}{N_{case}} - AF_{control}\left(\frac{N_{total}}{N_{case}}AF_{pop}\right) + AF_{control}^2(\frac{N_{control}}{N_{case}})}{AF_{control}[1 - \left(\frac{N_{total}}{N_{case}}AF_{total}\right)] + AF_{control}^2(\frac{N_{control}}{N_{case}})} \quad (6)$$

$$AF_{control}^2\left(\frac{N_{control}}{N_{case}}\right)OR + AF_{control}\left[1 - \left(\frac{N_{total}}{N_{case}}AF_{total}\right)\right]OR = AF_{control}^2\left(\frac{N_{control}}{N_{case}}\right) - AF_{control}\left(\frac{N_{control}}{N_{case}} + \frac{N_{total}}{N_{case}}AF_{total}\right) + \frac{N_{total}}{N_{case}}AF_{total} \quad (7)$$

Arrange as quadratic equation:

$$AF_{control}^2\left[\frac{N_{control}}{N_{case}}(OR - 1)\right] + AF_{control}\left[OR\left(1 - \left(\frac{N_{total}}{N_{case}}AF_{total}\right)\right) + \frac{1}{N_{case}}(N_{control} + N_{total}AF_{total})\right] - \frac{N_{total}}{N_{case}}AF_{total} = 0 \quad (8)$$

Let:

$$\left[a = \frac{N_{control}}{N_{case}}(OR - 1)\right] \quad (9)$$

$$b = \left[OR\left(1 - \left(\frac{N_{total}}{N_{case}}AF_{total}\right)\right) + \frac{1}{N_{case}}(N_{control} + N_{total}AF_{total})\right] \quad (10)$$

$$c = -\frac{N_{total}}{N_{case}}AF_{total} \quad (11)$$

$$AF_{control}^2 a + AF_{control} b + c = 0 \quad (12)$$

Choose the root greater than 0 and less than 1 to be $AF_{control}$ where x_1, x_2 are the roots

$$x_{1,2} = \frac{-b \pm \sqrt{b^2 - 4ac}}{2a} \quad (13)$$

$$AF_{control} = \begin{cases} x_1, & if\ 0 \leq x_1 \leq 1 \\ x_2, & otherwise \end{cases}$$

Use the calculated $AF_{control}$ to solve for AF_{case} where:

$$AF_{case} = \frac{N_{total}}{N_{case}}AF_{total} - \frac{N_{control}}{N_{case}}AF_{control} \quad (14)$$

Simulations show there is only one root [0,1].

Given the quadratic in equation (8) and the coefficients in (9 – 11), we sought to determine the possible solutions for the roots as shown in equation (13). Keeping N_{total} constant at 10,000, we examined 144 different scenarios with varying AF, OR, N_{case}, and $N_{control}$ as shown in the table below (all combinations and results in Table S1).

Parameter	Values
N_{case}	1000, 5000, 9000
$N_{control}$	9000, 5000, 1000
N_{total}	10000
AF_{total}	0, 0.1, 0.25, 0.5, 0.75, 1
OR	0.1, 0.4, 0.6, 0.8, 1.1, 1.2, 3, 5

We then used the values of the parameters to compute *a, b, c* as shown in equations (9-11), followed by calculation of the two roots using equation (13). Root 1 (x_1) and root 2 (x_2) were calculated as follows:

$$x_1 = \frac{-b - \sqrt{b^2 - 4ac}}{2a} \quad (15)$$

$$x_2 = \frac{-b + \sqrt{b^2 - 4ac}}{2a} \quad (16)$$

We observed the following results, indicating that for possible combinations of parameters, only one root (x_2) lies within [0,1]. Also note that when $a > 0$, $x_1 < 0$, and when $a < 0$, $x_1 > 1$.

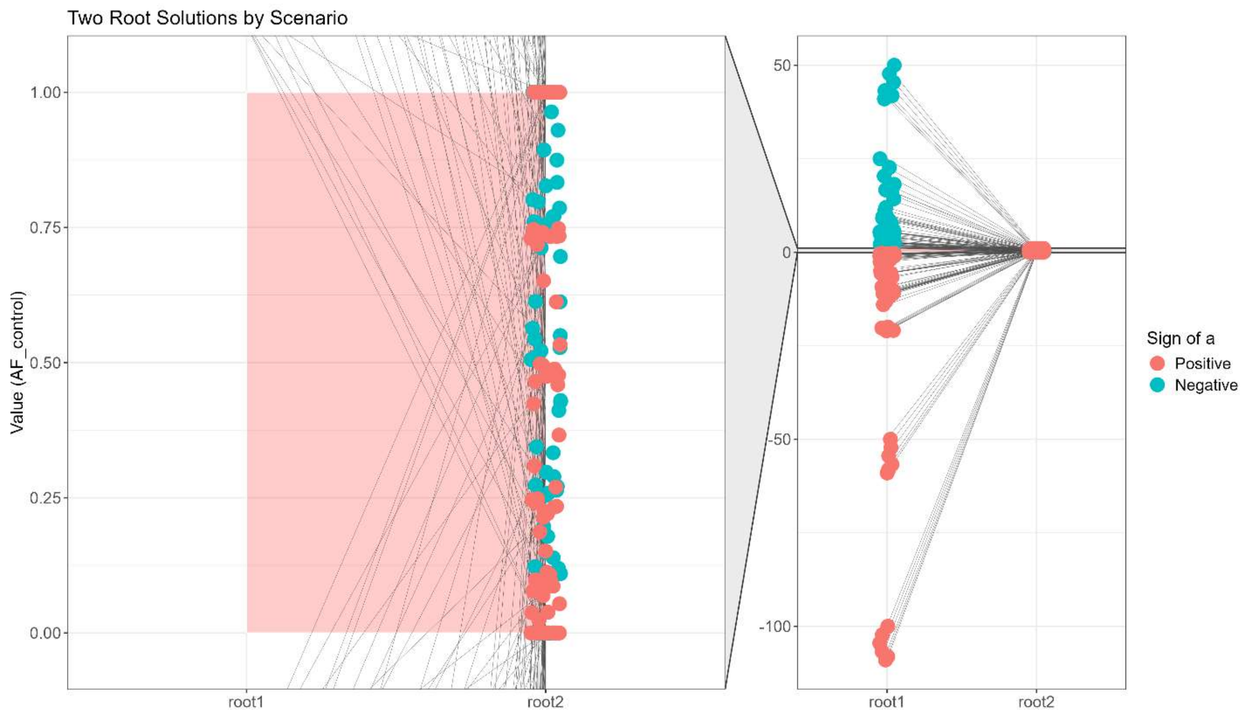

Figure S1. Simulations show only one root solves for valid control AF value. Simulations of 144 different scenarios with varying case and control sample size, OR, and AF were used to calculate the roots (two possible solutions for control AF). Root 2, calculated by equation (16) is always in the valid interval [0,1] (shown in the red rectangle), while root 1, calculated by equation (15) is always outside of the valid interval. We also note that when the coefficient *a* is positive, root 1 is lower than the valid interval (i.e. < 0) and when *a* is negative, root 1 is greater than the valid interval (i.e. > 1).

Case AF Derivation for Prostate Cancer GWAS

We selected a prostate cancer PGS based on a GWAS that published OR and control AF for each variant(Schumacher et al., 2018). Using the case and control sample sizes ($N_{case}, N_{control}$) we derive allele counts (AC) for the effect and non-effect alleles using $AC = AF * N$. We also know that the OR can be calculated using ACs as follows:

$$OR = \frac{AC_{effect,case} * AC_{non-effect,control}}{AC_{effect,control} * AC_{non-effect,case}} \tag{17}$$

We can then rearrange this equation to solve for the AC of the effect allele in cases, as needed for our reference data as follows:

$$AC_{effect,case} = \frac{OR * AC_{effect,control}}{1 - AC_{effect,control} + OR * AC_{effect,control}} \tag{18}$$

CaseControl_SE Bias Correction Framework

We observed systematic bias in the estimated case and control MAFs derived by CaseControl_SE in simulations with covariates and in real data. This bias was present in the estimated case, control, and total sample MAFs. To estimate this bias, we can use an estimate or proxy of the true MAF. We use

gnomAD MAFs as this proxy. Because the bias trends with MAF bin, we fit regression models by MAF bin. For each MAF bin ([0, 0.1) [0.1, 0.2) [0.2, 0.3) [0.3, 0.4) [0.4, 0.5]) we fit a second order polynomial where the outcome is the estimated MAF from the SE method and the predictor is the gnomAD MAF (Figure S2).

We evaluated using smaller (5%) MAF bins and observed nearly identical least squares (LS) difference. We therefore chose 10% MAFs for computational efficiency. To obtain the best fitting model for the majority of the data, we identified and excluded outliers defined as observations with an absolute value of the studentized residual > 3. We then refit the models without outliers. This results in the regression estimates shown below.

$$\widehat{MAF}_{CaseControl\_SE} = \hat{a} * MAF_{gnomAD}^{2} + \hat{b} * MAF_{gnomAD} + \hat{c} \tag{19}$$

The bias can then be estimated as:

$$\widehat{bias} = MAF_{gnomAD} - \widehat{MAF}_{CaseControl\_SE} \tag{20}$$

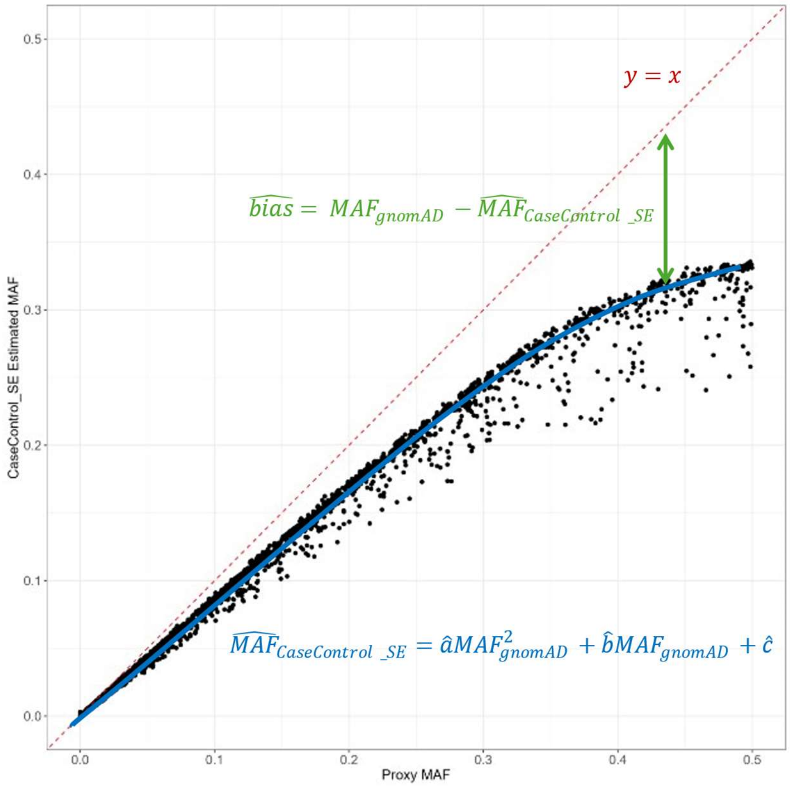

Figure S2. Bias correction framework to adjust CaseControl_SE estimates using gnomAD as proxies. The relationship between the proxy MAF (x-axis) and the estimated MAF (y-axis) is modeled through polynomial regressions (blue). This model is used to estimate the bias (green). The adjusted MAF estimate is estimated by adding the bias to the CaseControl_SE MAF output.

Since we observed a systematic bias where the estimated MAF was less than the true MAF, we estimate the bias by subtracting the predicted value from the gnomAD MAF. The adjusted MAF is calculated by adding the estimated bias to the estimated MAF output by CaseControl_SE. We found that using the same estimated bias for cases, controls, and total sample worked well.

$$MAF^*_{CaseControl\_SE} = MAF_{CaseControl\_SE} + \widehat{bias} \tag{21}$$

Bias adjustment for variants not in proxy data

To complete the bias adjustment for variants not present in the proxy data, the following steps are used:

1. Polynomial regression (second order) is fit using the proxy data (MAF_{gnomAD}) as the predictor and the CaseControl_SE MAF ($MAF_{CaseControl\_SE}$) as the outcome by gnomAD MAF bin, resulting in estimates for $\hat{a}, \hat{b}, \hat{c}$
2. For variants not in proxy dataset (i.e. CaseControl_SE MAF, but unknown gnomAD MAF), solve for the estimated gnomAD MAF ($\widehat{MAF}_{gnomAD}$) using $\hat{a}, \hat{b}, \hat{c}$

$$0 = \hat{a} * MAF^2_{gnomAD} + \hat{b} * MAF_{gnomAD} + (\hat{c} - MAF_{CaseControl\_SE}) \tag{22}$$

3. Calculate $\widehat{MAF}_{CaseControl\_SE}$

$$\widehat{MAF}_{CaseControl\_SE} = \hat{a} * \widehat{MAF}^2_{gnomAD} + \hat{b} * \widehat{MAF}_{gnomAD} + \hat{c} \tag{23}$$

4. Calculate $\widehat{bias}$:

$$\widehat{bias} = \widehat{MAF}_{gnomAD} - \widehat{MAF}_{CaseControl\_SE} \tag{24}$$

5. Calculate $MAF^*_{estimated}$ using equation (21)

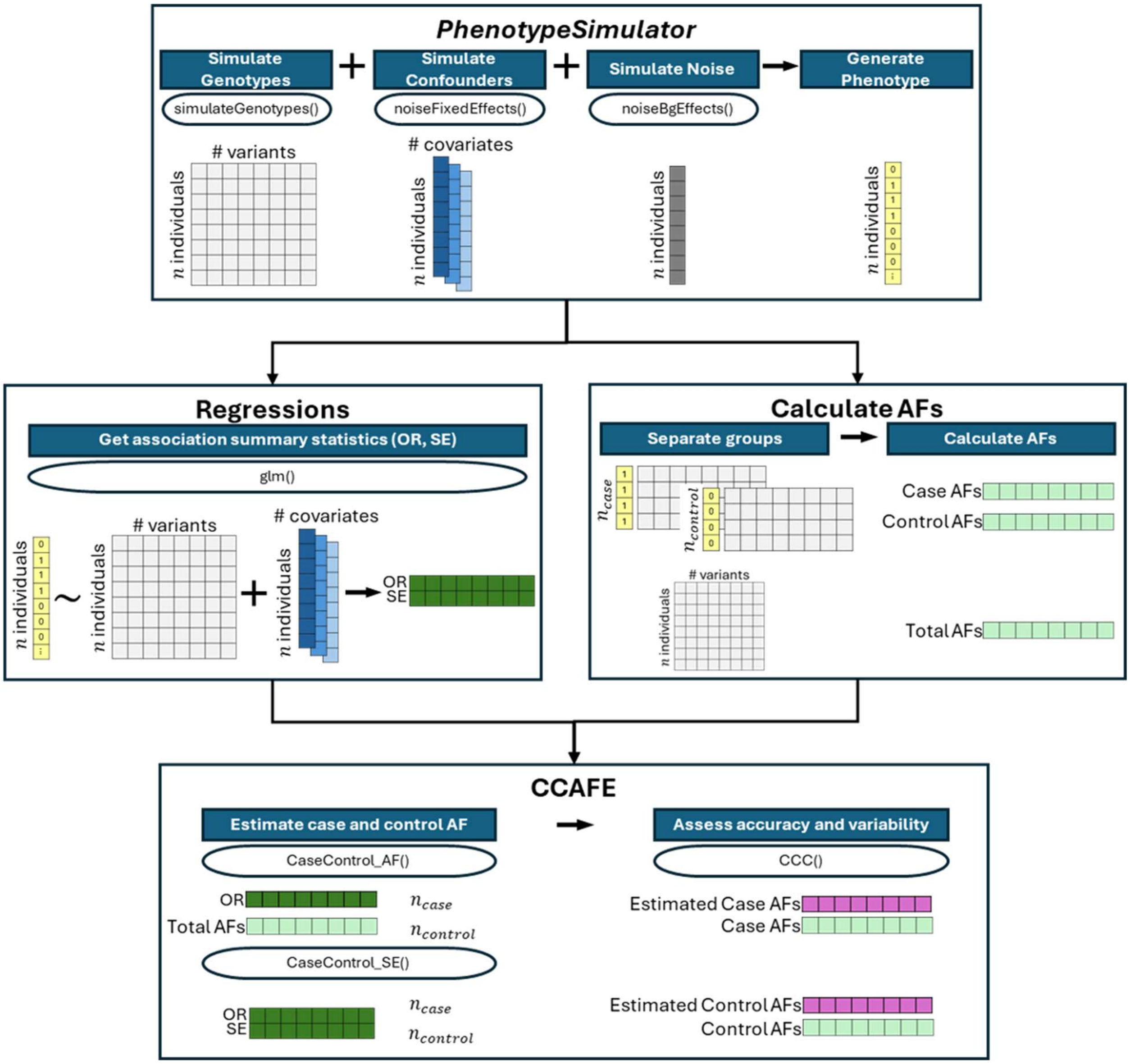

Figure S3. Simulation framework to assess *CCAFE* accuracy and variability. The *PhenotypeSimulator* R package was used to simulate genotypes, a binary phenotype, and covariates. Logistic regression was used on the simulated genotypes and phenotypes to obtain association summary statistics (OR, SE). The phenotype was also used to calculate AF for cases and controls. These simulations were used to test CaseControl_AF using the OR, total AF and case and control sample sizes as well as CaseControl_SE using the OR, SE, and case and control sample sizes. The output estimated case and control AFs were compared to the simulated case and control AFs using Lin's CCC.

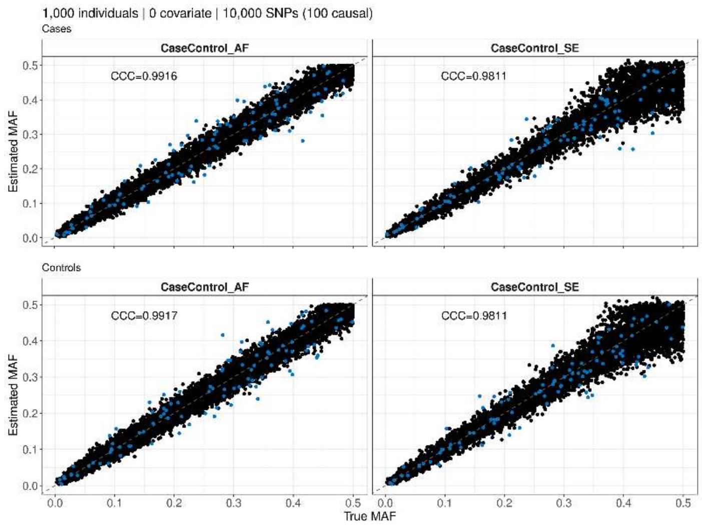

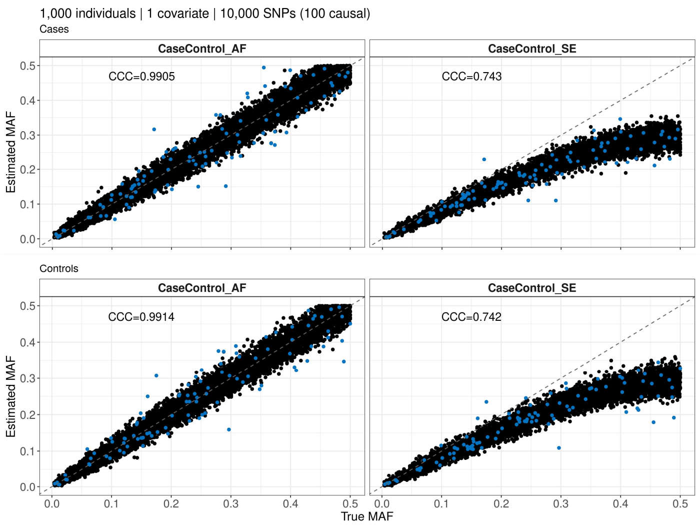

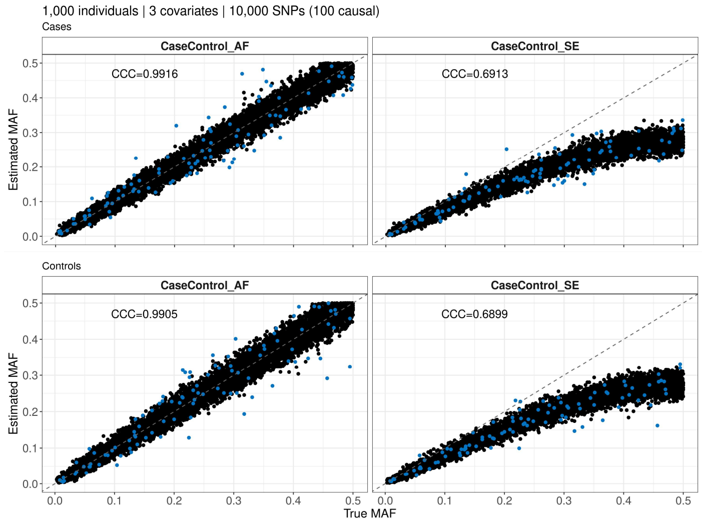

Figure S4. CaseControl_SE produces biased results in simulation when covariates included. Simulated genotypes and binary (case/control) phenotypes were generated using the PhenotypeSimulator R package for N=1,000 (500 cases and 500 controls). Genotypes for 10,000 SNPs, of which 100 were causal (blue), were generated for 1000 individuals. Logistic regression was used along with 0 (top left), 1 (top right), or 3 (bottom left) simulated covariates to generate summary statistics. The CCAFE R package was used to reconstruct the case and control AFs with total AF (left plots within each quadrant) or SE (right plots within each quadrant). Using the SE, bias was greater for higher MAFs and when more covariates were included. Using total AF was accurate across the simulation parameters evaluated. CCC values are reported in Table 1.

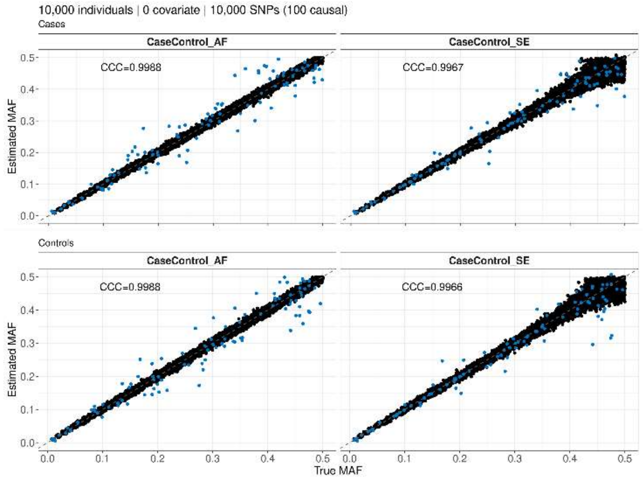

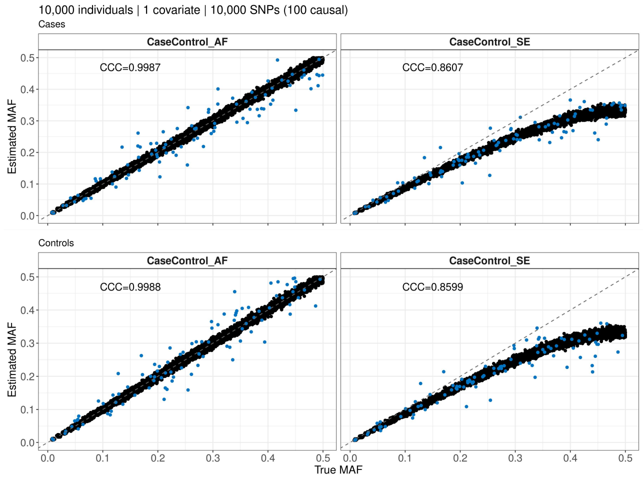

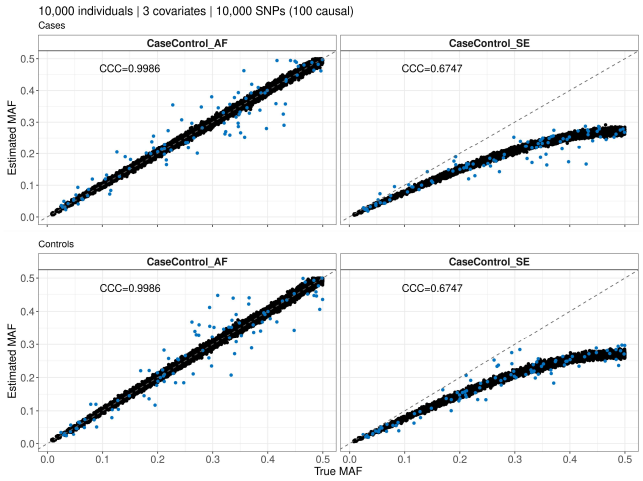

Figure S5. CaseControl_SE produces biased results in simulation when covariates included. Simulated genotypes and binary (case/control) phenotypes were generated using the PhenotypeSimulator R package for N=10,000 (5000 cases and 5000 controls). Genotypes for 10,000 SNPs, of which 100 were causal (blue), were generated for 1000 individuals. Logistic regression was used along with 0 (top left), 1 (top right), or 3 (bottom left) simulated covariates to generate summary statistics. The CCAFE R package was used to reconstruct the case and control AFs with total AF (left plots within each quadrant) or SE (right plots within each quadrant). Using the SE, bias was greater for higher MAFs and when more covariates were included. Using total AF was accurate across the simulation parameters evaluated. CCC values are reported in Table 1.

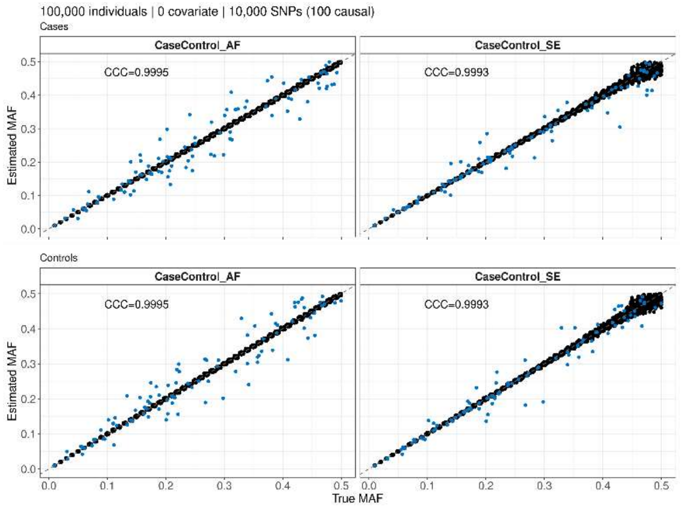

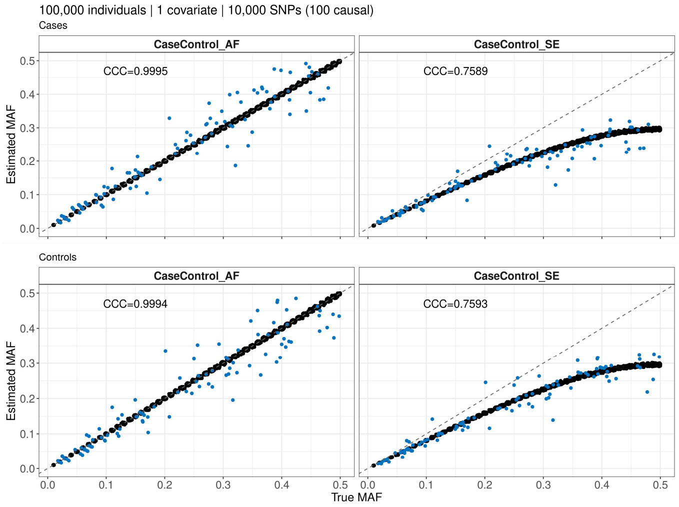

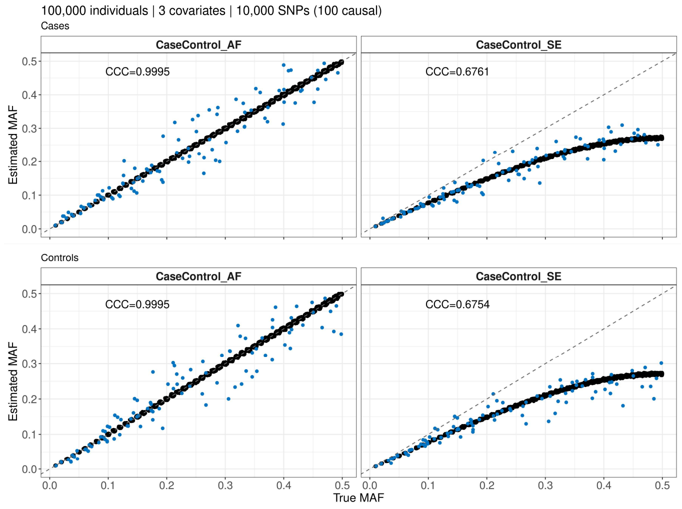

Figure S6. CaseControl_SE produces biased results in simulation when covariates included. Simulated genotypes and binary (case/control) phenotypes were generated using the PhenotypeSimulator R package for N=100,000 (50,000 cases and 50,000 controls). Genotypes for 10,000 SNPs, of which 100 were causal (blue), were generated for 1000 individuals. Logistic regression was used along with 0 (top left), 1 (top right), or 3 (bottom left) simulated covariates to generate summary statistics. The CCAFE R package was used to reconstruct the case and control AFs with total AF (left plots within each quadrant) or SE (right plots within each quadrant). Using the SE, bias was greater for higher MAFs and when more covariates were included. Using total AF was accurate across the simulation parameters evaluated. CCC values are reported in Table 1.

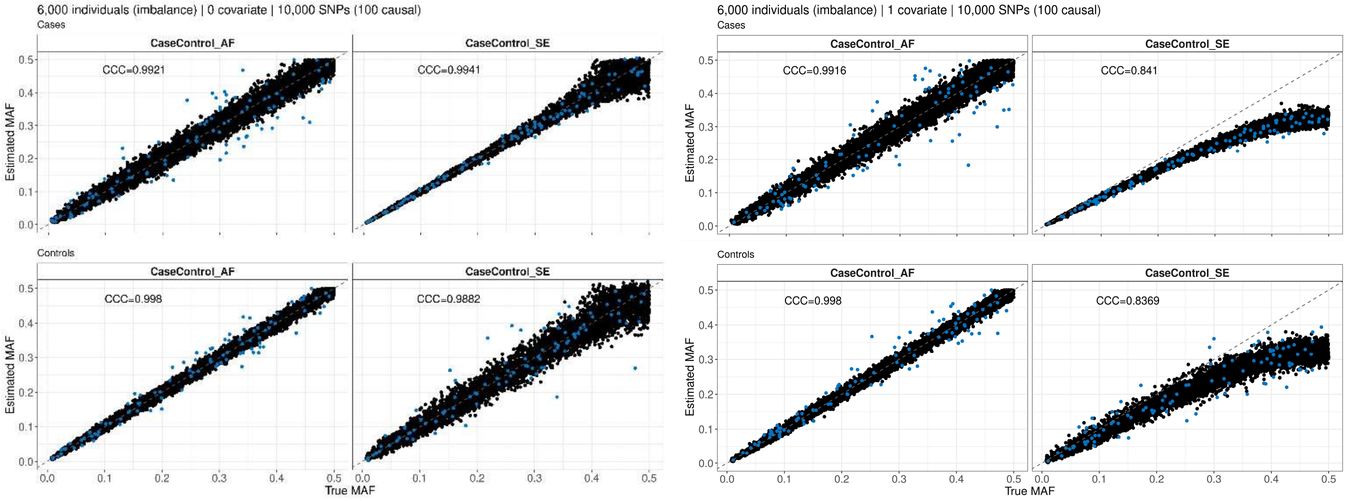

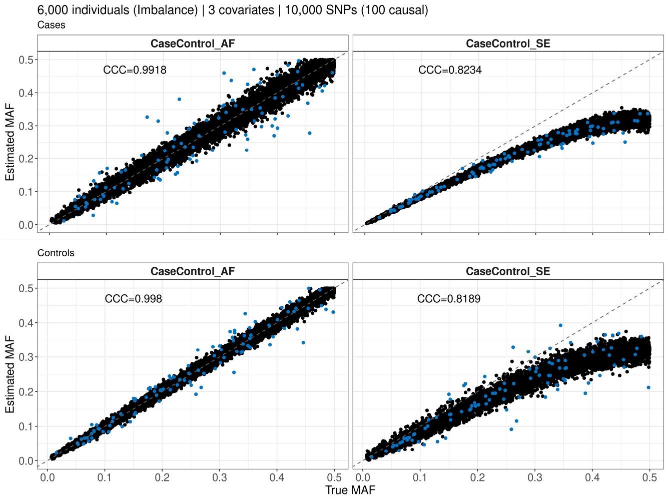

Figure S7. CaseControl_SE produces biased results in simulation when covariates included and sample imbalance. Simulated genotypes and binary (case/control) phenotypes were generated using the PhenotypeSimulator R package for N=6,000 (600 cases and 5,400 controls). Genotypes for 10,000 SNPs, of which 100 were causal (blue), were generated for 1000 individuals. Logistic regression was used along with 0 (top left), 1 (top right), or 3 (bottom left) simulated covariates to generate summary statistics. The CCAFE R package was used to reconstruct the case and control AFs with total AF (left plots within each quadrant) or SE (right plots within each quadrant). Using the SE, bias was greater for higher MAFs and when more covariates were included. Using total AF was accurate across the simulation parameters evaluated. CCC values are reported in Table 1.

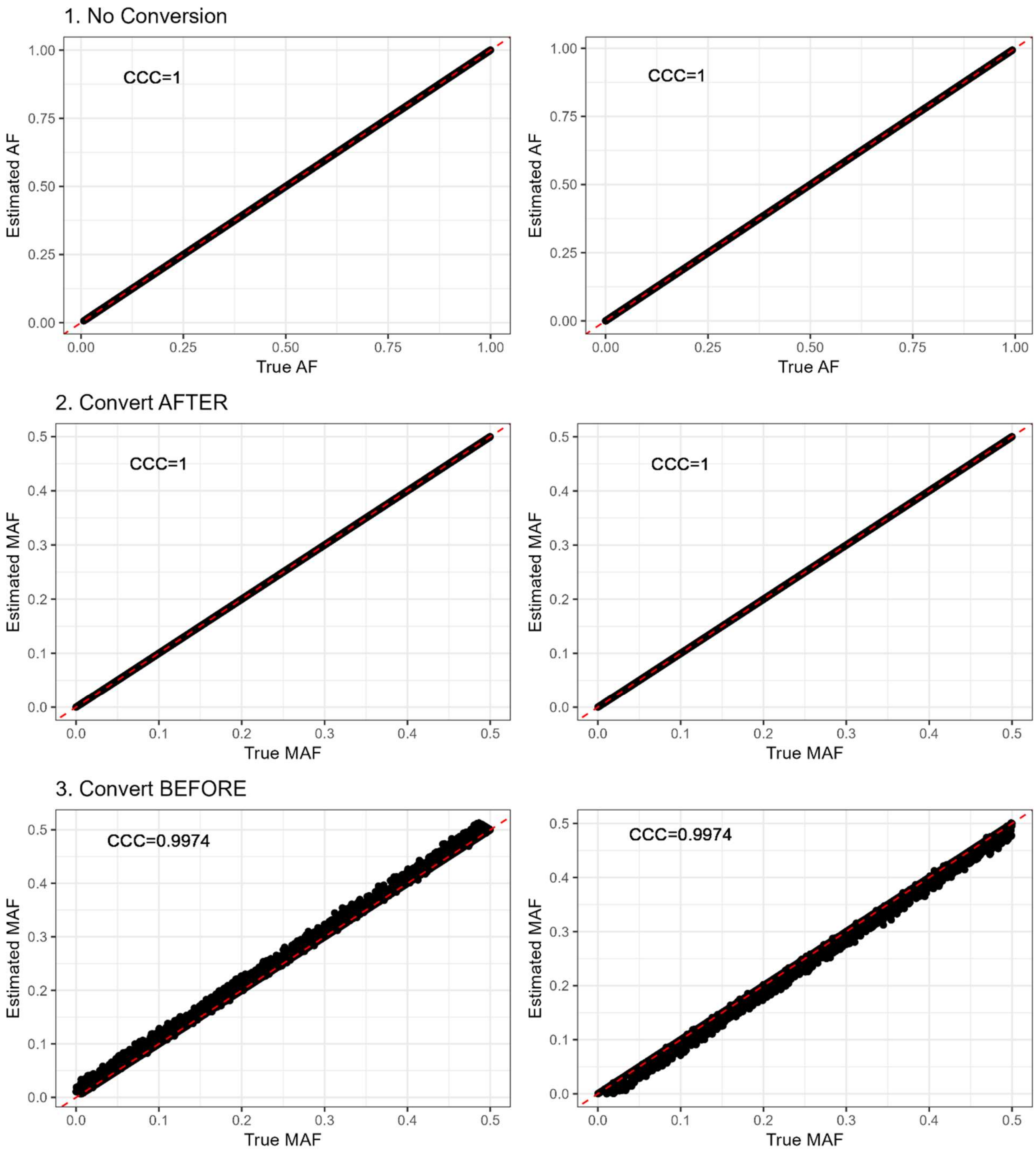

Figure S8. Converting AF to MAF increases variability in case and control estimates using CaseControl_AF. Using 2400 simulated variants three scenarios were tested to estimate case AF (Left) and control AF (Right) using CaseControl_AF: 1) using the total AF to estimate case and control AFs 2) using total AF to estimate case and control AFs then converting to minor AF (MAF) 3) converting total AF to MAF and estimating case and control MAFs. Converting first to MAF introduces variability. Lin's CCC is reported between the true and estimated AF.

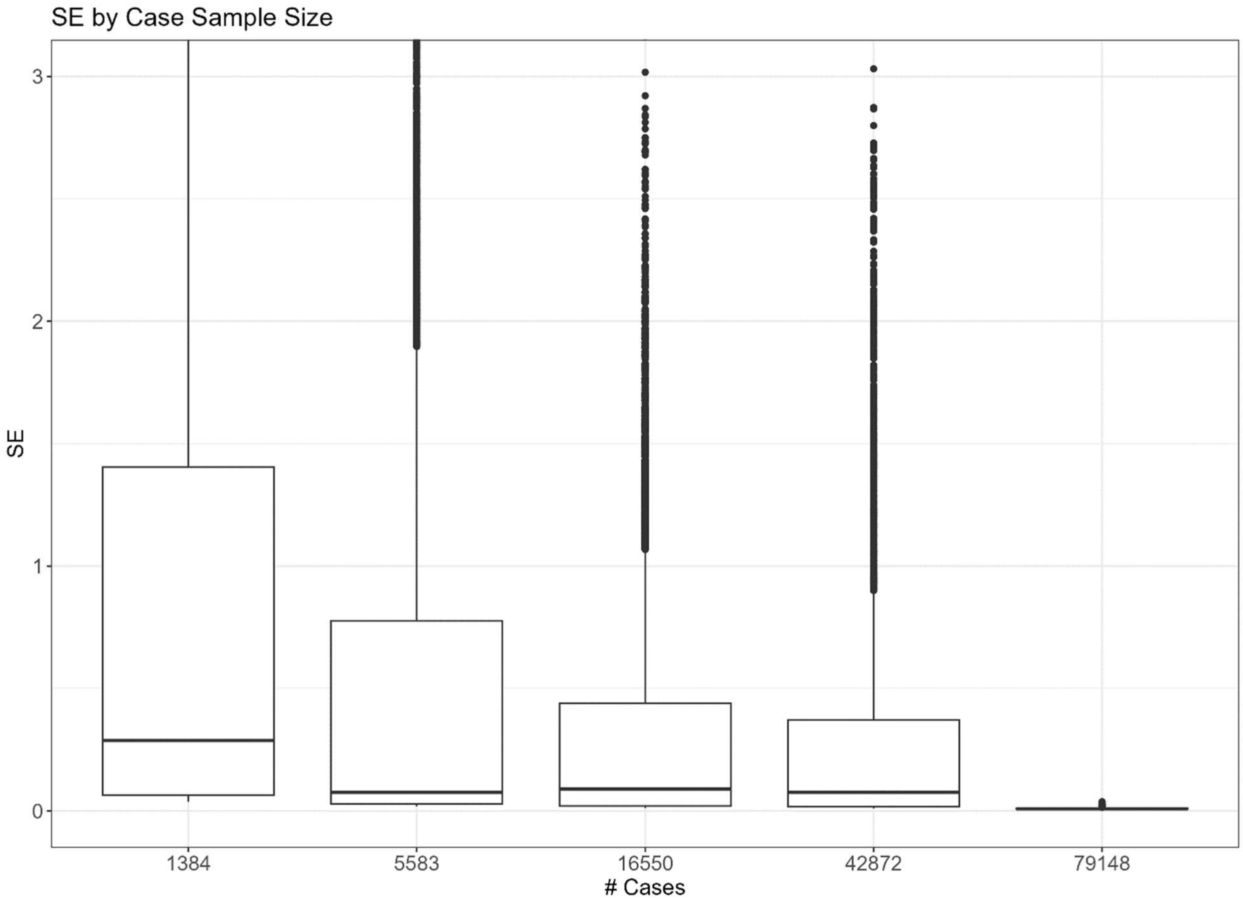

Figure S9. Larger sample sizes have smaller SE and less variability in SE. Here we examined the relationship between the case sample size and the standard error. We see studies with larger case sample sizes have a smaller SE including the minimum observed SE. Additionally, larger case sample sizes result in less variability in the SE across the variants. For each boxplot the center line shows the median SE and the upper and lower hinge represent the 75th and 25th percentile, respectively. The upper and lower whiskers are the largest and smallest values no more than 1.5* interquartile range (IQR) from the hinge.

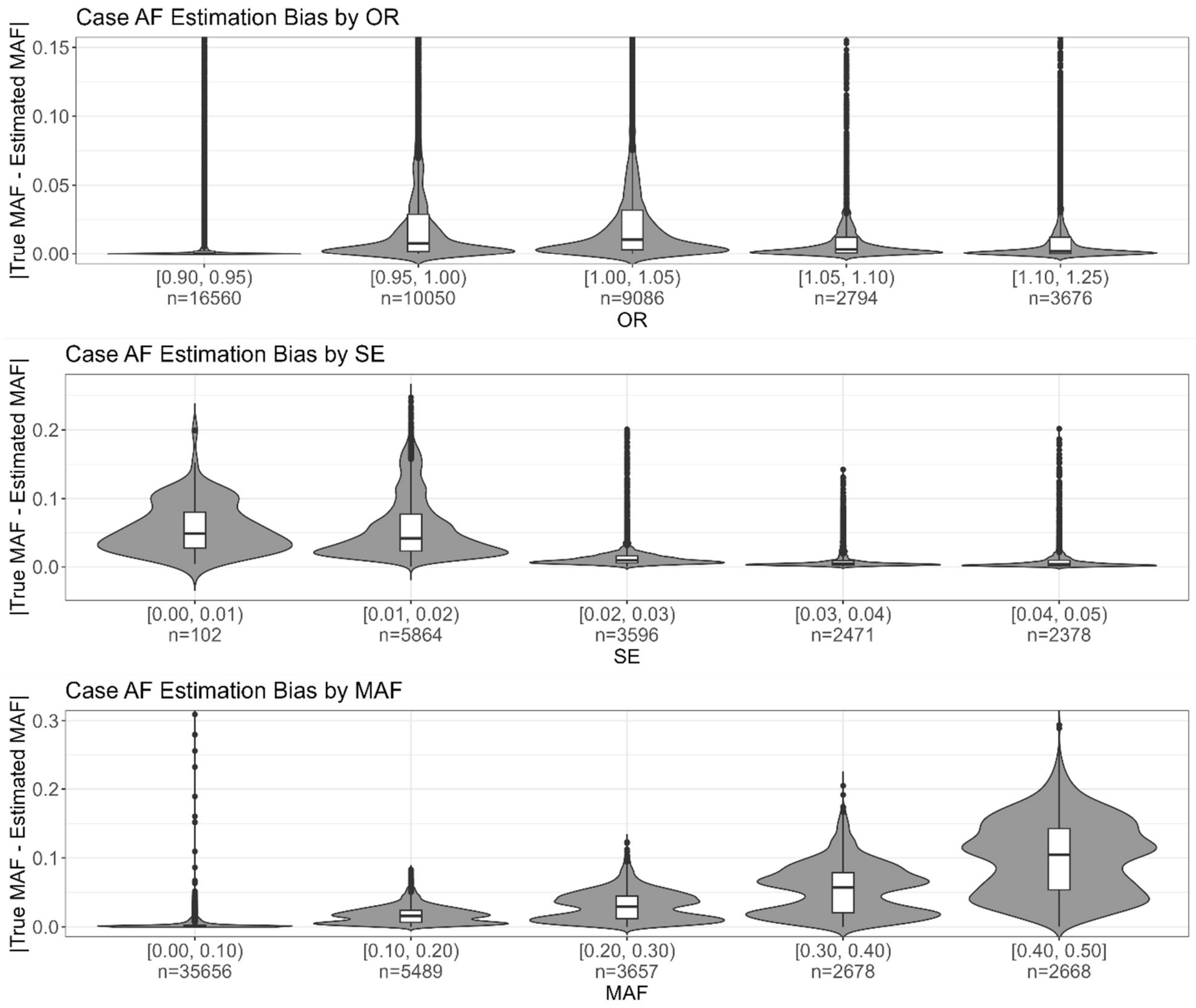

Figure S10. CaseControl_SE has high bias at large MAF and small SE. The difference between the true MAF and CaseControl_SE estimated MAF is compared across different bins for the OR (top), SE (middle), and MAF (bottom). Here the results for all datasets shown in Figure S7 are aggregated and plotted, with the number of SNPs in each bin shown on the x-axis. We see that bias is higher for smaller SE and larger MAF

Table S1. Simulations show only one possible root (root2) falls within valid interval [0,1] for control AF solution										
**OR**	**AF**	**N_case**	**N_control**	**N_total**	**a**	**b**	**c**	**root1**	**root2**	**sim**
0.1	0	5000	5000	10000	-0.9000	1.1000	0.0000	1.2222	0.0000	1
0.4	0	5000	5000	10000	-0.6000	1.4000	0.0000	2.3333	0.0000	2
0.6	0	5000	5000	10000	-0.4000	1.6000	0.0000	4.0000	0.0000	3
0.8	0	5000	5000	10000	-0.2000	1.8000	0.0000	9.0000	0.0000	4
0.1	0	9000	1000	10000	-0.1000	0.2111	0.0000	2.1111	0.0000	5
0.4	0	9000	1000	10000	-0.0667	0.5111	0.0000	7.6667	0.0000	6
0.6	0	9000	1000	10000	-0.0444	0.7111	0.0000	16.0000	0.0000	7
0.8	0	9000	1000	10000	-0.0222	0.9111	0.0000	41.0000	0.0000	8
0.1	0	1000	9000	10000	-8.1000	9.1000	0.0000	1.1235	0.0000	9
0.4	0	1000	9000	10000	-5.4000	9.4000	0.0000	1.7407	0.0000	10
0.6	0	1000	9000	10000	-3.6000	9.6000	0.0000	2.6667	0.0000	11
0.8	0	1000	9000	10000	-1.8000	9.8000	0.0000	5.4444	0.0000	12
0.1	0.1	5000	5000	10000	-0.9000	1.2800	-0.2000	1.2435	0.1787	13
0.4	0.1	5000	5000	10000	-0.6000	1.5200	-0.2000	2.3941	0.1392	14
0.6	0.1	5000	5000	10000	-0.4000	1.6800	-0.2000	4.0774	0.1226	15
0.8	0.1	5000	5000	10000	-0.2000	1.8400	-0.2000	9.0900	0.1100	16
0.1	0.1	9000	1000	10000	-0.1000	0.3111	-0.1111	2.6995	0.4116	17
0.4	0.1	9000	1000	10000	-0.0667	0.5778	-0.1111	8.4699	0.1968	18
0.6	0.1	9000	1000	10000	-0.0444	0.7556	-0.1111	16.8516	0.1484	19
0.8	0.1	9000	1000	10000	-0.0222	0.9333	-0.1111	41.8806	0.1194	20
0.1	0.1	1000	9000	10000	-8.1000	10.0000	-1.0000	1.1248	0.1098	21
0.4	0.1	1000	9000	10000	-5.4000	10.0000	-1.0000	1.7458	0.1061	22
0.6	0.1	1000	9000	10000	-3.6000	10.0000	-1.0000	2.6739	0.1039	23
0.8	0.1	1000	9000	10000	-1.8000	10.0000	-1.0000	5.4537	0.1019	24
0.1	0.25	5000	5000	10000	-0.9000	1.5500	-0.5000	1.2923	0.4299	25
0.4	0.25	5000	5000	10000	-0.6000	1.7000	-0.5000	2.5000	0.3333	26
0.6	0.25	5000	5000	10000	-0.4000	1.8000	-0.5000	4.2026	0.2974	27
0.8	0.25	5000	5000	10000	-0.2000	1.9000	-0.5000	9.2291	0.2709	28
0.1	0.25	9000	1000	10000	-0.1000	0.4611	-0.2778	3.8986	0.7125	29
0.4	0.25	9000	1000	10000	-0.0667	0.6778	-0.2778	9.7388	0.4278	30
0.6	0.25	9000	1000	10000	-0.0444	0.8222	-0.2778	18.1558	0.3442	31
0.8	0.25	9000	1000	10000	-0.0222	0.9667	-0.2778	43.2107	0.2893	32
0.1	0.25	1000	9000	10000	-8.1000	11.3500	-2.5000	1.1275	0.2737	33
0.4	0.25	1000	9000	10000	-5.4000	10.9000	-2.5000	1.7547	0.2638	34
0.6	0.25	1000	9000	10000	-3.6000	10.6000	-2.5000	2.6859	0.2586	35
0.8	0.25	1000	9000	10000	-1.8000	10.3000	-2.5000	5.4682	0.2540	36
0.1	0.5	5000	5000	10000	-0.9000	2.0000	-1.0000	1.4625	0.7597	37
0.4	0.5	5000	5000	10000	-0.6000	2.0000	-1.0000	2.7208	0.6126	38
0.6	0.5	5000	5000	10000	-0.4000	2.0000	-1.0000	4.4365	0.5635	39
0.8	0.5	5000	5000	10000	-0.2000	2.0000	-1.0000	9.4721	0.5279	40
0.1	0.5	9000	1000	10000	-0.1000	0.7111	-0.5556	6.2176	0.8935	41

0.4	0.5	9000	1000	10000	-0.0667	0.8444	-0.5556	11.9705	0.6962	42
0.6	0.5	9000	1000	10000	-0.0444	0.9333	-0.5556	20.3869	0.6131	43
0.8	0.5	9000	1000	10000	-0.0222	1.0222	-0.5556	45.4499	0.5501	44
0.1	0.5	1000	9000	10000	-8.1000	13.6000	-5.0000	1.1353	0.5437	45
0.4	0.5	1000	9000	10000	-5.4000	12.4000	-5.0000	1.7745	0.5218	46
0.6	0.5	1000	9000	10000	-3.6000	11.6000	-5.0000	2.7097	0.5126	47
0.8	0.5	1000	9000	10000	-1.8000	10.8000	-5.0000	5.4944	0.5056	48
0.1	0.75	5000	5000	10000	-0.9000	2.4500	-1.5000	1.7923	0.9299	49
0.4	0.75	5000	5000	10000	-0.6000	2.3000	-1.5000	3.0000	0.8333	50
0.6	0.75	5000	5000	10000	-0.4000	2.2000	-1.5000	4.7026	0.7974	51
0.8	0.75	5000	5000	10000	-0.2000	2.1000	-1.5000	9.7291	0.7709	52
0.1	0.75	9000	1000	10000	-0.1000	0.9611	-0.8333	8.6474	0.9637	53
0.4	0.75	9000	1000	10000	-0.0667	1.0111	-0.8333	14.2921	0.8746	54
0.6	0.75	9000	1000	10000	-0.0444	1.0444	-0.8333	22.6730	0.8270	55
0.8	0.75	9000	1000	10000	-0.0222	1.0778	-0.8333	47.7141	0.7859	56
0.1	0.75	1000	9000	10000	-8.1000	15.8500	-7.5000	1.1554	0.8014	57
0.4	0.75	1000	9000	10000	-5.4000	13.9000	-7.5000	1.8043	0.7698	58
0.6	0.75	1000	9000	10000	-3.6000	12.6000	-7.5000	2.7395	0.7605	59
0.8	0.75	1000	9000	10000	-1.8000	11.3000	-7.5000	5.5234	0.7544	60
0.1	1	5000	5000	10000	-0.9000	2.9000	-2.0000	2.2222	1.0000	61
0.4	1	5000	5000	10000	-0.6000	2.6000	-2.0000	3.3333	1.0000	62
0.6	1	5000	5000	10000	-0.4000	2.4000	-2.0000	5.0000	1.0000	63
0.8	1	5000	5000	10000	-0.2000	2.2000	-2.0000	10.0000	1.0000	64
0.1	1	9000	1000	10000	-0.1000	1.2111	-1.1111	11.1111	1.0000	65
0.4	1	9000	1000	10000	-0.0667	1.1778	-1.1111	16.6667	1.0000	66
0.6	1	9000	1000	10000	-0.0444	1.1556	-1.1111	25.0000	1.0000	67
0.8	1	9000	1000	10000	-0.0222	1.1333	-1.1111	50.0000	1.0000	68
0.1	1	1000	9000	10000	-8.1000	18.1000	-10.0000	1.2346	1.0000	69
0.4	1	1000	9000	10000	-5.4000	15.4000	-10.0000	1.8519	1.0000	70
0.6	1	1000	9000	10000	-3.6000	13.6000	-10.0000	2.7778	1.0000	71
0.8	1	1000	9000	10000	-1.8000	11.8000	-10.0000	5.5556	1.0000	72
1.1	0	5000	5000	10000	0.1000	2.1000	0.0000	-21.0000	0.0000	73
1.2	0	5000	5000	10000	0.2000	2.2000	0.0000	-11.0000	0.0000	74
3	0	5000	5000	10000	2.0000	4.0000	0.0000	-2.0000	0.0000	75
5	0	5000	5000	10000	4.0000	6.0000	0.0000	-1.5000	0.0000	76
1.1	0	9000	1000	10000	0.0111	1.2111	0.0000	-109.0000	0.0000	77
1.2	0	9000	1000	10000	0.0222	1.3111	0.0000	-59.0000	0.0000	78
3	0	9000	1000	10000	0.2222	3.1111	0.0000	-14.0000	0.0000	79
5	0	9000	1000	10000	0.4444	5.1111	0.0000	-11.5000	0.0000	80
1.1	0	1000	9000	10000	0.9000	10.1000	0.0000	-11.2222	0.0000	81
1.2	0	1000	9000	10000	1.8000	10.2000	0.0000	-5.6667	0.0000	82
3	0	1000	9000	10000	18.0000	12.0000	0.0000	-0.6667	0.0000	83
5	0	1000	9000	10000	36.0000	14.0000	0.0000	-0.3889	0.0000	84

1.1	0.1	5000	5000	10000	0.1000	2.0800	-0.2000	-20.8957	0.0957	85
1.2	0.1	5000	5000	10000	0.2000	2.1600	-0.2000	-10.8918	0.0918	86
3	0.1	5000	5000	10000	2.0000	3.6000	-0.2000	-1.8539	0.0539	87
5	0.1	5000	5000	10000	4.0000	5.2000	-0.2000	-1.3374	0.0374	88
1.1	0.1	9000	1000	10000	0.0111	1.2000	-0.1111	-108.0925	0.0925	89
1.2	0.1	9000	1000	10000	0.0222	1.2889	-0.1111	-58.0861	0.0861	90
3	0.1	9000	1000	10000	0.2222	2.8889	-0.1111	-13.0383	0.0383	91
5	0.1	9000	1000	10000	0.4444	4.6667	-0.1111	-10.5238	0.0238	92
1.1	0.1	1000	9000	10000	0.9000	10.0000	-1.0000	-11.2102	0.0991	93
1.2	0.1	1000	9000	10000	1.8000	10.0000	-1.0000	-5.6538	0.0983	94
3	0.1	1000	9000	10000	18.0000	10.0000	-1.0000	-0.6421	0.0865	95
5	0.1	1000	9000	10000	36.0000	10.0000	-1.0000	-0.3558	0.0781	96
1.1	0.25	5000	5000	10000	0.1000	2.0500	-0.5000	-20.7411	0.2411	97
1.2	0.25	5000	5000	10000	0.2000	2.1000	-0.5000	-10.7329	0.2329	98
3	0.25	5000	5000	10000	2.0000	3.0000	-0.5000	-1.6514	0.1514	99
5	0.25	5000	5000	10000	4.0000	4.0000	-0.5000	-1.1124	0.1124	100
1.1	0.25	9000	1000	10000	0.0111	1.1833	-0.2778	-106.7342	0.2342	101
1.2	0.25	9000	1000	10000	0.0222	1.2556	-0.2778	-56.7204	0.2204	102
3	0.25	9000	1000	10000	0.2222	2.5556	-0.2778	-11.6077	0.1077	103
5	0.25	9000	1000	10000	0.4444	4.0000	-0.2778	-9.0689	0.0689	104
1.1	0.25	1000	9000	10000	0.9000	9.8500	-2.5000	-11.1926	0.2482	105
1.2	0.25	1000	9000	10000	1.8000	9.7000	-2.5000	-5.6353	0.2465	106
3	0.25	1000	9000	10000	18.0000	7.0000	-2.5000	-0.6148	0.2259	107
5	0.25	1000	9000	10000	36.0000	4.0000	-2.5000	-0.3249	0.2138	108
1.1	0.5	5000	5000	10000	0.1000	2.0000	-1.0000	-20.4881	0.4881	109
1.2	0.5	5000	5000	10000	0.2000	2.0000	-1.0000	-10.4772	0.4772	110
3	0.5	5000	5000	10000	2.0000	2.0000	-1.0000	-1.3660	0.3660	111
5	0.5	5000	5000	10000	4.0000	2.0000	-1.0000	-0.8090	0.3090	112
1.1	0.5	9000	1000	10000	0.0111	1.1556	-0.5556	-104.4786	0.4786	113
1.2	0.5	9000	1000	10000	0.0222	1.2000	-0.5556	-54.4591	0.4591	114
3	0.5	9000	1000	10000	0.2222	2.0000	-0.5556	-9.2697	0.2697	115
5	0.5	9000	1000	10000	0.4444	2.8889	-0.5556	-6.6869	0.1869	116
1.1	0.5	1000	9000	10000	0.9000	9.6000	-5.0000	-11.1643	0.4976	117
1.2	0.5	1000	9000	10000	1.8000	9.2000	-5.0000	-5.6066	0.4955	118
3	0.5	1000	9000	10000	18.0000	2.0000	-5.0000	-0.5855	0.4744	119
5	0.5	1000	9000	10000	36.0000	-6.0000	-5.0000	-0.2985	0.4652	120
1.1	0.75	5000	5000	10000	0.1000	1.9500	-1.5000	-20.2411	0.7411	121
1.2	0.75	5000	5000	10000	0.2000	1.9000	-1.5000	-10.2329	0.7329	122
3	0.75	5000	5000	10000	2.0000	1.0000	-1.5000	-1.1514	0.6514	123
5	0.75	5000	5000	10000	4.0000	0.0000	-1.5000	-0.6124	0.6124	124
1.1	0.75	9000	1000	10000	0.0111	1.1278	-0.8333	-102.2336	0.7336	125
1.2	0.75	9000	1000	10000	0.0222	1.1444	-0.8333	-52.2181	0.7181	126
3	0.75	9000	1000	10000	0.2222	1.4444	-0.8333	-7.0332	0.5332	127

5	0.75	9000	1000	10000	0.4444	1.7778	-0.8333	-4.4238	0.4238	128
1.1	0.75	1000	9000	10000	0.9000	9.3500	-7.5000	-11.1371	0.7482	129
1.2	0.75	1000	9000	10000	1.8000	8.7000	-7.5000	-5.5800	0.7467	130
3	0.75	1000	9000	10000	18.0000	-3.0000	-7.5000	-0.5675	0.7342	131
5	0.75	1000	9000	10000	36.0000	-16.0000	-7.5000	-0.2854	0.7299	132
1.1	1	5000	5000	10000	0.1000	1.9000	-2.0000	-20.0000	1.0000	133
1.2	1	5000	5000	10000	0.2000	1.8000	-2.0000	-10.0000	1.0000	134
3	1	5000	5000	10000	2.0000	0.0000	-2.0000	-1.0000	1.0000	135
5	1	5000	5000	10000	4.0000	-2.0000	-2.0000	-0.5000	1.0000	136
1.1	1	9000	1000	10000	0.0111	1.1000	-1.1111	-100.0000	1.0000	137
1.2	1	9000	1000	10000	0.0222	1.0889	-1.1111	-50.0000	1.0000	138
3	1	9000	1000	10000	0.2222	0.8889	-1.1111	-5.0000	1.0000	139
5	1	9000	1000	10000	0.4444	0.6667	-1.1111	-2.5000	1.0000	140
1.1	1	1000	9000	10000	0.9000	9.1000	-10.0000	-11.1111	1.0000	141
1.2	1	1000	9000	10000	1.8000	8.2000	-10.0000	-5.5556	1.0000	142
3	1	1000	9000	10000	18.0000	-8.0000	-10.0000	-0.5556	1.0000	143
5	1	1000	9000	10000	36.0000	-26.0000	-10.0000	-0.2778	1.0000	144
